# Effect of Simple Oral Dental Extraction on Systemic Serum Amyloid A Concentrations in Horses

**DOI:** 10.1002/vms3.70104

**Published:** 2024-11-07

**Authors:** Amelia E. Sidwell, Marco Duz, Adeel Khan, Ronald Bodnàr, Sam Luis Hole

**Affiliations:** ^1^ School of Veterinary Medicine and Science University of Nottingham Nottingham UK; ^2^ Pool House Equine Hospital IVC Evidensia Lichfield UK

**Keywords:** exodontia, periodontal disease, serum amyloid A, tooth extraction

## Abstract

**Background:**

The translocation of gingival commensals resulting in measurable systemic inflammation has been described in humans and non‐equine veterinary species with dental disorders, particularly periodontal disease. Routine odontoplasty does not result in increased serum amyloid A (SAA) concentration in horses, but a measurable increase in SAA concentration in horses undergoing dental extractions could suggest that local inflammation resulting from more severe dental disease has potential for wider, systemic consequences that warrants further study.

**Objectives:**

To determine whether SAA increases in horses undergoing simple, oral extraction of non‐fractured cheek teeth with and without periodontal disease.

**Study Design:**

Prospective cohort study.

**Methods:**

SAA was measured using a stall‐side test in horses undergoing simple oral extraction of cheek teeth with intact clinical crowns at baseline (*T* = 0), 24 h (*T* = 24) and 48 h (*T* = 48) post‐extraction.

**Results:**

Eight horses and 4 ponies aged between 4 and 23 years underwent cheek tooth extraction. A statistically significant difference in SAA concentration was noted between groups with and without periodontal disease at both 24 h (*p* = 0.004) and 48 h (*p* = 0.043). At 24 h, the median SAA concentration was 135 mg/L (range: 0–260 mg/L; IQR: 77.5–174 mg/L) in horses with periodontal disease and 27.5 mg/L (range: 0–47 mg/L; IQR: 4.8–43.5 mg/L) in horses without periodontal disease. At 48 h, median SAA concentration was 264 mg/L (range: 236–440 mg/L; IQR: 245.5–300.5 mg/L) in horses with periodontal disease and 0 mg/L (range = 0–41 mg/L; IQR: 0–21.8 mg/L) in horses without periodontal disease.

**Main Limitations:**

Small sample group. Horses undergoing extraction of fractured cheek teeth were not included.

**Conclusions:**

Extraction of non‐fractured cheek teeth does not result in a remarkable increase in SAA, except in horses with periodontal disease. These results suggest that periodontal disease in horses is associated with a local inflammatory response, which in turn drives the development of systemic inflammation, resulting in detectable increases in inflammatory markers when diseased periodontal tissues are disturbed.

## Graphical abstract

Extraction of non‐fractured cheek teeth does not result in a remarkable increase in SAA, except in horses with periodontal disease. Median SAA concentration at 24 h was 135 mg/L and 27.5 mg/L in horses with and without periodontal disease, respectively. At 48 h, median SAA concentration was 264 mg/L in horses with, and 0 mg/L in horses without periodontal disease, suggesting that periodontal disease in horses is associated with detectable increases in inflammatory markers when diseased periodontal tissues are disturbed.

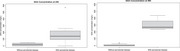

## 1 Introduction

Similarly to other species, transient bacteraemia has been proven to occur in horses undergoing exodontia (Kern et al. 2017). However, the consequences of this are not known. In addition to bacteraemia (Martins et al. 2023), humans undergoing exodontia also demonstrate a measurable, systemic inflammatory response, with increases seen in serum fibrinogen and C‐reactive protein concentrations (Graziani et al. 2017). Increases in circulating serum amyloid A (SAA) are described in mice with endodontic lesions, and concentrations of inflammatory cytokines are associated with the severity of dental disease in cats (Hirai et al. 2019; Watanabe et al. 2019).

Periodontal disease (PD) in humans has been implicated in the pathogenesis of systemic inflammation with associations reported between PD and other inflammatory conditions, including arthritis, type 2 diabetes mellitus and vascular disease (Martínez‐García and Hernández‐Lemus 2021). In dogs, significant associations have been highlighted between PD and pathology in other organs, including the liver parenchyma, kidney and myocardium (DeBowes et al. 1996), although this may be a consequence bacteraemia associated with translocated gingival commensals, which in humans has been linked to infective endocarditis (Lockhart et al. 2023). Bacteraemia is more likely to develop during oral surgery involving infected dental tissues (Takai et al. 2005), as would be the case in the majority of equine dental extractions, save extractions of vestigial first premolars (‘wolf teeth’). Whilst there appear to be no apparent adverse consequences as a sequela to transient bacteraemia following exodontia in horses, even in the absence of antibiotic administration (Kern et al. 2017), the occurrence of a systemic inflammatory response with the potential to impact other organ systems is not yet established in this species.

SAA is a major acute phase protein (APP) in horses. Tissue trauma due to infection, inflammation or injury results in the release of pro‐inflammatory mediators, such as interleukin‐1, ‐6 and tumour necrosis factor alpha (TNF‐*α*) ultimately stimulating the hepatic synthesis of SAA (Cray, Zaias, and Altman 2009; Haltmayer, Schwendenwein, and Licka 2017; Petersen, Nielsen, and Heegaard 2004). Increases in plasma SAA concentrations can be seen as little as 6 h after the onset of the acute phase response (APR), with peak values typically seen at 48 h (Haltmayer, Schwendenwein, and Licka 2017; Jacobsen and Andersen 2010; Petersen, Nielsen, and Heegaard 2004). The rapid increase and decrease in SAA seen in response to an inflammatory stimulus make it a valuable tool for monitoring clinical progression (Belgrave et al. 2013). Although non‐specific, SAA is considered a sensitive marker of both septic and non‐septic inflammatory conditions in adult horses (Belgrave et al. 2013; Jacobsen and Andersen 2010; Hoeberg et al. 2022).

Localised concentrations of pro‐inflammatory cytokines correlate with clinical progression and help evaluate treatment outcomes in humans with dental disease, including PD, pulpitis and periapical lesions (Hirai et al. 2019; Rechenberg, Galicia, and Peters 2016; Reis et al. 2014). Subgingival plaque samples have been used to assess the equine oral microbiome (Kennedy et al. 2016); the sampling gingival crevicular fluid (GCF) is likely more suitable for determining inflammatory markers (Guentsch et al. 2011). However, this method has not yet been described in horses and is likely more time‐consuming and technically challenging than the relative ease of peripheral blood sampling.

Increased SAA concentrations are not seen in horses undergoing routine odontoplasty, even in cases where iatrogenic trauma occurred (Birmingham and Mason 2019).

This study aims to determine whether horses undergoing simple oral extraction of cheek teeth (CT) with an intact clinical crown display increases in SAA concentrations in peripheral blood, which may relate to the degree of the bacteraemia and inflammatory response associated with tooth extraction in this species. Additionally, a comparison was made between horses with and without concurrent PD to assess the potential impact of this dental pathology on systemic inflammation.

## 2 Materials and Methods

### 2.1 Inclusion Criteria

All clinically healthy, adult horses (> 4 years of age) undergoing standard, simple oral extraction of CT with an intact clinical crown at a specialist dental referral hospital in the United Kingdom between 2021 and 2023 were eligible for inclusion. Exclusion criteria included known or suspected comorbidities, including pituitary pars intermedia dysfunction (PPID), horses having received any medication within the 14 days prior to exodontia or dental fracture pre‐ or intra‐exodontia.

The study was completed with the approval of The Committee for Animal Research and Ethics (CARE) of the School of Veterinary Medicine and Science at the University of Nottingham.

### 2.2 Classification of Periodontal Disease

Horses were included in the group with PD if the reason for dental extraction was advanced PD (estimated > 50% attachment loss) or horses undergoing concurrent treatment for early to moderate PD (estimated 25%–50% attachment loss), involving diastema widening and professional cleaning (Klugh 2005). Horses with mild gingivitis (< 5 mm probing depth) where only odontoplasty (routine ‘rasping’ of overgrowths and sharp enamel points) were not considered for inclusion in this group. All diagnoses were made by an EVDC specialist in equine dentistry based on oroscopic and radiographic findings.

### 2.3 Pre‐Operative Treatment and Surgery

All CT were extracted per os by an experienced veterinary surgeon (EVDC specialist or resident in training) using a standard technique of gingival elevation, molar spreading, oscillation with forceps and extraction with forceps and a fulcrum (Dixon et al. 2010). Exodontia was performed under standing chemical restraint with a detomidine continuous rate infusion (30 mg detomidine in 500 mL Hartmann's solution, yielding a solution of 0.06 mg/mL), titrated to effect via a 15‐drop/mL giving set. Locoregional anaesthesia was performed in all cases. Either a maxillary or mandibular (inferior alveolar) nerve block was performed, depending on the tooth to be extracted, with 2% mepivacaine hydrochloride. Inferior alveolar nerve blocks were performed via a transcutaneous, extraoral approach at the level of the mandibular foramen using an 18 gauge 25 cm spinal needle (Rice 2017). The maxillary nerve was anaesthetised using a caudolateral approach as it enters the pterygopalatine fossa at the maxillary foramen using an 18 gauge 9 cm spinal needle (Tremaine 2019). Intra‐oral, local anaesthesia of the soft tissues (gingiva and coronal aspects of the periodontal ligament) surrounding the tooth was administered using 2% lidocaine hydrochloride with epinephrine (Lignospan Special, Septodont).

All patients received phenylbutazone (4.4 mg/kg IV, Equipalazone, Dechra) and oxytetracycline (5 mg/kg IV, Engemycin DD, MSD) between 30 and 60 min prior to starting the extraction (with the surgical start time defined as when initial gingival elevation was performed). Patients 1–3 received butorphanol tartrate (0.02 mg/kg IV, Torphadine, Dechra) and patients 4–12 received morphine (0.1 mg/kg IV) at the start of the procedure.

### 2.4 Post‐Operative Management

All horses remained hospitalised for a minimum of 3 days post‐procedure, during which they remained on strict box rest and fed a diet of soft soaked hay, fed from the floor. Horses were not exercised. Horses remained on injectable antibiotics (oxytetracycline 5 mg/kg IV SID, Engemycin DD, MSD) and non‐steroidal anti‐inflammatories (phenylbutazone, 4.4 mg/kg IV SID, Equipalazone, Dechra) for a total of 3 days, prior to transitioning onto oral anti‐inflammatories (phenylbutazone 2.2 mg/kg PO BID, Equipalazone, Dechra). On day 3, all horses were sedated with detomidine (0.01 mg/kg IV, Domidine, Dechra) and butorphanol tartrate (0.02 mg/kg IV, Torphadine, Dechra) to replace the oral packing with vinyl polysiloxane (VPS). All alveoli were examined oroscopically at this time and appeared to be healthy, with no evidence of complication.

### 2.5 SAA Samples and Assay

Blood samples utilised for this study include leftover blood from samples obtained to monitor the patient perioperatively following the prolonged administration of alpha‐2 agonists to undertake standing surgical procedures. Samples were obtained at the following intervals: before the commencement of oral extraction (*T* = 0), and at 24 h (*T* = 24) and 48 h (*T* = 48) post‐procedure.

Blood samples were collected through a jugular venous catheter and SAA was measured immediately from whole blood using a point‐of‐care SAA assay (StableLab EQ‐1, Zoetis, UK) marketed for use in horses.

### 2.6 Data Analysis

Power calculation was performed after samples were obtained from pilot data noted a trend towards increased SAA in horses undergoing exodontia due to PDor in those undergoing concurrent periodontal treatment (diastema widening, professional cleaning) alongside dental extractions. Power calculations were performed using G*Power (version 3.1.9.6). When divided into two groups (horses with and without PD), six horses per group were necessary to complete a study with a power of 0.95 and an error probability (alpha) of only 0.05.

Descriptive statistics for each group were calculated and reported as median, minimum and maximum values of SAA concentration. Considering the small sample size, a non‐parametric test such as Mann–Whitney was used to formally compare the SAA concentrations between the two groups. Any difference was considered as statistically significant where *p* < 0.05. Analysis was performed using Rv 4.2.1, R Core Team (2023).

## 3 Results

### 3.1 Study Population and Samples

A total of 12 patients: 8 horses (3 warmbloods, 1 thoroughbred, 1 Irish draught, 2 cobs, 1 mixed breed and 1 Appaloosa) and 4 ponies (1 Shetland and 3 Welsh Section A) met the inclusion criteria. The median age was 13.5 years (range 4–23). Mares were under‐represented, with a total of 8 geldings and 4 mares included in the study.

Reasons for cheek tooth extraction were infundibular caries (*n* = 2), periapical infection (*n* = 4) and PD (*n* = 6). In addition to the five horses undergoing exodontia for severe PD (patients number 3, 6, 9, 10 and 11), a further horse undergoing extraction for advanced infundibular caries (patient number 5) underwent concurrent treatment for diastemata and PD at the time of extraction, resulting in a total of 6 horses undergoing treatment for PD. In the horses without PD, two had additional procedures performed. One horse had infundibular restorations on a further carious cheek tooth and one horse underwent concurrent sinus trephination and sinoscopy (patients 4 and 2, respectively). The clinical findings for each case and SAA concentration at each time point are summarised in Table 1.

**TABLE 1:** Patient details and reasons for exodontia with SAA values at baseline (*T* = 0), 24 (*T* = 24) and 48 h (*T* = 48). Horses with periodontal disease are highlighted in bold.

Patient	Signalment	Group	Procedure	*T* = 0	*T* = 24	*T* = 48	Notes
1	10‐year‐old WB gelding	No PD	X 209	0	47	29	
2	21‐year‐old Shetland gelding	No PD	X 209; SIN/TRP	12	36	0	Concurrent sinus trephination and sinoscopy
**3**	**23‐year‐old Appaloosa gelding**	**PD**	**X 407**	**0**	**127**	**259**	**407 extracted due to periodontal disease. Periodontal treatment (professional cleaning)**
4	14‐year‐old Unspecified/mixed breed mare	No PD	X 209; R/C 208	0	0	0	Concurrent restorations performed on CT 208
**5**	**13‐year‐old Welsh Section A X gelding**	**PD**	**X 106; X 206; D/ODY 306/07/08**	**0**	**0**	**269**	**Concurrent diastema widening and periodontal treatment (professional cleaning)**
**6**	**6‐year‐old Welsh Section A mare**	**PD**	**X 408**	**0**	**184**	**236**	**408 extracted due to periodontal disease. Concurrent periodontal treatment (professional cleaning)**
7	16‐year‐old Cob mare	No PD	X 106 × 107; R/C 109, 209	0	0	0	Concurrent restorations (109, 209)
8	4‐year‐old Welsh Section A gelding	No PD	X 407	0	46	0	Routine odontoplasty performed concurrently.
**9**	**16‐year‐old TB gelding**	**PD**	**X 310; D/ODY 410/411**	**78**	**260**	**311**	**310 extracted due to periodontal disease. Diastema widening and periodontal treatment (professional cleaning)**
**10**	**6‐year‐old ISH gelding**	**PD**	**X 308; D/ODY 309/310/311; D/ODY 407/40; D/ODY 409/410/411; PRO; ODY 106, 206, 109, 209**	**0**	**144**	**440**	**308 extracted due to periodontal disease. Diastema widening and periodontal treatment (professional cleaning)**
**11**	**18‐year‐old Belgian Warmblood gelding**	**PD**	**X 108; X 109**	**0**	**61**	**241**	**108 and 109 extracted due to periodontal disease**.
12	7‐year‐old Warmblood gelding	No PD	X 107	0	19	41	Periapical infection with maxillary swelling.

Relevant AVDC/EVDC abbreviations: D/ODY—diastema widening; PRO—professional cleaning; R/C—restoration (composite); SIN/TRP—sinus trephination; X—simple extraction (Available at: https://www.vetdentdms.org/forms/abbreviations.pdf).

**FIGURE 1:**
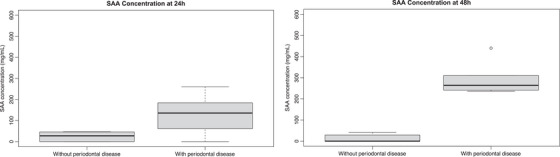
(a,b) Box plots demonstrating the range and median serum amyloid A concentrations in horses with and without periodontal disease at 24 and 48 h.

A statistically significant difference was noted between groups with and without PD at both 24 h (*p* = 0.004) and 48 h (*p* = 0.043) (Figure 1). At 24 h, the median SAA concentrations were 135 mg/L (range: 0–260 mg/L; IQR: 77.5–174 mg/L) in horses with PD and 27.5 mg/L (range: 0–47 mg/L; IQR: 4.8–43.5 mg/L) in horses without PD. At 48 h, median SAA concentration was 264 mg/L (range: 236–440 mg/L; IQR: 245.5–300.5 mg/L) in horses with PD and 0 mg/L (range = 0–41 mg/L; IQR: 0–21.8 mg/L) in horses without PD.

## 4 Discussion

Extraction of CT with an intact clinical crown did not result in a statistically significant increase in SAA, unless the indication for extraction was PD or the patient underwent concurrent treatment for PD.

Indications for dental extraction in horses include periapical infection, dental fracture, severe caries and PD (Earley, Rawlinson, and Baratt 2013). In humans and companion animals, PD is amongst the most common indications for exodontia (O'Neill et al. 2014, 2021; Wallis and Holcombe 2020). Increases in inflammatory cytokines are seen in cats undergoing dental extractions and are associated with the presence of significant tooth resorption and dental fractures (Watanabe et al. 2019). Increased SAA is seen in humans with chronic periodontitis and apical periodontitis (Peeran et al. 2020; Hirai et al. 2019). Whilst terminology between equine and human dental conditions is not always consistent, apical periodontitis in humans is most often the result of endodontic disease (Arias et al. 2023). This would be considered analogous with periapical infection in horses, which frequently occurs as sequelae to endodontic disease (Rowley et al. 2022).

One limitation of this study was a failure to include fractured CT, hence the presence of a systemic inflammatory response during extraction cannot be directly compared to that of other veterinary species. However, in contrast to humans with apical periodontitis teeth with a periapical infection, horses in this study undergoing exodontia due to periapical infection did not demonstrate a measurable increase in systemic SAA concentration.

Improvements in diagnostic imaging technology, coupled with a greater understanding of radiographic changes associated with early periapical infections (Townsend et al. 2011), and increased engagement of horse owners in prophylactic dental healthcare may mean teeth with evidence of endodontic disease or periapical infection are being diagnosed and extracted at an earlier stage, prior to the development of significant localised infection. This may offer some explanation as to why these horses did not show any detectable signs of systemic inflammation associated with exodontia in contrast to humans, where exodontia is only likely to be performed where other treatments for endodontic disease are unfeasible or have failed.

However, in this study, teeth were extracted based on radiographic findings consistent with a periapical infection (Townsend et al. 2011), which is less sensitive than other methods for detecting endodontic disease (Liuti, Smith, and Dixon 2018). It would therefore perhaps be expected that these teeth were at a more advanced stage of disease, and therefore more likely to elicit an inflammatory response. Additionally, horses with concurrent facial swelling or sinusitis (patients 12 and 2, respectively) also failed to produce marked increases in SAA concentrations, nor did SAA concentration appear to be significantly influenced by concurrent surgical or dental procedures in horses without PD (Pollock et al. 2005; Jacobsen et al. 2009).

These findings may support a hypothesis that it is the involvement of certain bacterial species in dental disease that results in greater local inflammation and thus a greater systemic inflammatory response. Despite limitations in culturing oral bacteria, bacterial populations appear to vary with the presence or absence of certain dental pathologies in horses (Borkent and Kennedy 2022). No assessment of the microbiome was performed in any of the horses included in the study to determine which pathogens may be associated with a greater systemic inflammatory response, but it is understood that certain bacteria are capable of modulating the host immune response in human PD (Dixon, Bainbridge, and Darveau 2004).

PD is an inflammatory condition initiated by an immune response to microorganisms within a dental plaque, a biofilm of microorganisms embedded in a polymer matrix (Marsh 2006). Bacteria within this biofilm trigger a local inflammatory response through the interaction between pathogen‐associated molecular patterns (PAMPs) and pattern‐recognition receptors (PRRs) in host cells, and by inducing the infiltration of inflammatory cells (Galler et al. 2021). PAMPs, such as lipopolysaccharide (LPS), activate macrophages to secrete pro‐inflammatory molecules, including cytokines, tumour necrosis factor, prostaglandins and hydrolytic enzymes (Page 1991). Activation of PRRs on vascular endothelial cells also disrupts vascular homeostasis through the activation of coagulation and fibrinolytic systems and increasing endothelial permeability (Li, Ouyang, and Lin 2022).

Initially believed to occur as secondary disease process, as sequelae to feed impaction or disorders of dental wear and eruption, the presence of dental plaque and the presence of certain microbial populations appear to be associated with PD in horses (Cox, Dixon, and Smith 2012; Kennedy et al. 2016). Further, histological features of equine PD would also suggest a pathogenesis similar to that seen in other species, with the presence of neutrophilic inflammation of the gingival epithelium and infiltration of mononuclear cells and eosinophils within the lamina propria and submucosa indicative of a localised inflammatory process (Cox, Dixon, and Smith 2012).

*Prevotella* and *Fusobacterium* spp. are dominant gram negative obligate anaerobes present in the oral microbiome of horses with dental disease (Bienert et al. 2003; Kern et al. 2017) and high numbers of these bacteria have been associated with PD in humans (Dixon, Bainbridge, and Darveau 2004). This shift in the oral microbial composition in certain diseased states likely results in an increased local inflammatory response through the release of LPS (Kennedy et al. 2016; Kim et al. 2021; Murray and Wilton 2003).

PD is associated with increases in systemic inflammatory markers in humans, with control of local infection resulting in a reduction in serum inflammatory markers (D'Aiuto et al. 2004). It is the complex nature of host–pathogen interactions in PD that may explain the increased SAA seen in horses with PD, and not those undergoing exodontia for other reasons. However, baseline SAA concentrations in horses with PD suggest that there was no detectable systemic response until periodontal tissues were manipulated during dental treatments. As such, systemic inflammatory markers appear to be an unsuitable tool for the detection of PD in horses and cannot substitute a comprehensive oral examination to fully evaluate and prognosticate PD in the equine patient (Collins and Dixon 2005).

This limits the potential applications of SAA concentrations in peripheral blood samples to monitor the resolution of inflammation after periodontal treatment or extraction. However, a significant limitation of this study was the inability to follow up of SAA concentrations beyond 48 h to determine a pattern of resolution.

It must be noted that two horses from each group (patients 2 and 9) had slightly elevated baseline SAA concentrations. Considering the inclusion and exclusion criteria, any pre‐existing inflammatory processes other than dental disease were subclinical. Further parameters, such as total cell count, may have helped determine the significance of these values and the overall results of this study.

Of all dental procedures, extraction has the highest incidence of bacteraemia in human patients, followed by root scaling and root planning (Martins et al. 2023). Bacteraemia has also been demonstrated in horses undergoing exodontia, with isolated organisms generally corresponding those cultured from extracted teeth (Kern et al. 2017). Blood cultures were not performed in this study, so conclusions cannot be made as to whether the presence or degree of bacteraemia correlates with elevations in SAA concentrations.

Horses included in the study received pre‐operative antimicrobials and non‐steroidal anti‐inflammatory drugs, which could influence SAA (Busk, Jacobsen, and Martinussen 2010). However, human patients undergoing third molar extraction display a measurable systemic inflammatory response despite this (Graziani et al. 2017).

Further research is warranted to better establish a link between the presence and extent of bacteraemia with the development of a systemic inflammatory response in horses undergoing dental procedures, with and without antibiotic administration, to better guide treatment and promote antimicrobial stewardship. Additionally, it must be established whether certain oral pathogens stimulate a more robust inflammatory response.

There are some limitations with the point‐of‐care (POC) SAA assay used. The test can be influenced by sample type, product batch, conditions such as hyperlipaemia or haemolysis and a hook effect is reported at higher SAA concentrations (Kiemle et al. 2022). Cartridges from the same batch were used in this study and the same methodology was applied to all samples. Additionally, the SAA concentrations obtained in this study (< 3000 mg/L) are unlikely to be significantly affected by the hook effect.

The results of this study suggest that the disturbance of locally inflamed tissues in cases of equine PD is associated with a detectable increase in SAA concentrations. These findings appear to support the existing literature, that PD results in a significant host inflammatory response, and may suggest that controlling local inflammation is a vital step in managing PD in horses. Further research is needed into the equine oral microbiome and its effect on local immunity and inflammatory processes in diseased states.

## Author Contributions

Amelia E. Sidwell and Ronald Bodnàr conceived the original idea and study design. Amelia E. Sidwell and Sam L. Hole were involved in data collection. Amelia E. Sidwell wrote initial drafts of the manuscript. Marco Duz obtained necessary ethical approval and performed data analysis. All authors were involved in editing the final manuscript.

## Ethics Statement

The study was completed with the approval of Committee for Animal Research and Ethics (CARE) at the School of Veterinary Medicine and Science, University of Nottingham (Project Number 3977231018).

## Conflicts of Interest

The authors declare no conflicts of interest.

### Peer Review

The peer review history for this article is available at https://publons.com/publon/10.1002/vms3.70104

## Data Availability

The data that support the findings of this study are available from the corresponding author upon reasonable request.
